# Sex-determining Region Y-box transcription factor 13 promotes breast cancer cell proliferation and glycolysis by activating the tripartite motif containing 11-mediated Wnt/β-catenin signaling pathway

**DOI:** 10.1080/21655979.2022.2073127

**Published:** 2022-05-25

**Authors:** Xiaoyan Jin, Xuan Shao, Wenyang Pang, Zhengyi Wang, Jian Huang

**Affiliations:** a Department of Breast Surgery, Second Affiliated Hospital of Zhejiang University School of Medicine, Hangzhou, Zhejiang Province, China; b Department of Breast Surgery, Taizhou Municipal Hospital, Taizhou, Zhejiang Province, China

**Keywords:** Breast cancer, SOX13, glycolysis, TRIM11, wnt/β-catenin signaling pathway

## Abstract

Breast cancer is the most frequent cancer among women and the second highest mortality in female across the world. Recent studies have illustrated that sex-determining region Y (SRY)-box protein (SOX) family plays essential roles in regulating various cancers. Nevertheless, the detailed effects of SOX13 on breast cancer are still uncovered. In our present study, SOX13 protein level was measured by using western blot assay in tissues and cells, and the results showed that SOX13 was upregulated in breast cancer tissues and cells compared with normal samples. Moreover, silencing SOX13 inhibited breast cancer cell viability, arrested cell cycle at G1/S phase and suppressed glycolysis, while overexpression of SOX13 reversed these events. Additionally, SOX13 knockdown reduced the level of proteins related to Wnt/β-catenin signaling pathway, whereas overexpression of tripartite motif containing 11 (TRM11) efficiently attenuated the effects, indicating that SOX13 controlled Wnt/β-catenin pathway depending on TRIM11. Furthermore, the data gained from xenograft tumor model illustrated that silencing SOX13 suppressed the tumor growth in nude mice and the glycolysis of tissues. In conclusion, our investigation illustrated that SOX13 facilitated breast cancer cell proliferation and glycolysis by modulating Wnt/β-catenin signaling pathway affected via TRIM11.

## Graphical abstract:This investigation revealed that SOX13 promoted breast cancer proliferation and glycolysis *in vivo* and *in vitro*. Additionally, SOX13 was able to regulate Wnt/β-catenin signaling pathway by mediating TRIM11 expression

**Figure:**
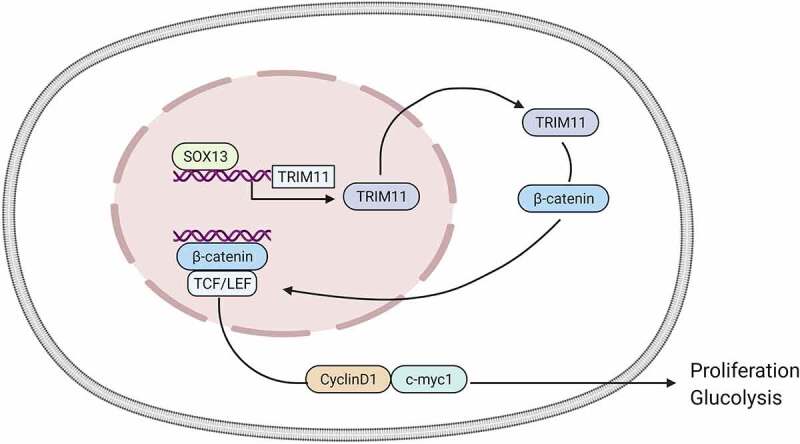

## Highlight

- This work elucidated the roles of SOX13 in breast cancer progression.
- This study uncovered that SOX13 promoted breast cancer growth and glycolysis.
- The finding proved that SOX13 regulated TRIM11/Wnt/β-catenin signaling pathway.
- More downstream genes of SOX13 involved in glycolysis will be explored.

## Introduction

Breast cancer, a highly heterogeneous disease, is considered the leading malignancy in women all over the world and the second highest cause of mortality in female [1–3]. Neither early diagnosis nor treatment for a favorable prognosis of breast cancer has been effective in reducing mortality [4]. Surgery as the major treatment strategy for treating breast cancer has some negative effects on patients’ physiology, emotion, as well as social activity [5]. Emerging evidence proves that breast carcinogenesis is a complicated process with elusive molecular mechanisms [6]. In addition, several vital oncogenes and anti-oncogenes have been found to be associated with breast tumorigenesis [7,8]. However, the details of pathogenic mechanisms and therapeutic gene targets remain unclear. For good prognosis, it is crucial to explore novel genes related to breast cancer progress.

Even though cancer cells are rich in oxygen, glucose metabolism is still largely dependent on glycolysis. Aerobic glycolysis, also known as the Warburg effect, is verified to be the leading outcome of oncogenic drivers [9]. Wnt/β-catenin signaling pathway is essential for maintaining the development of normal tissue and disordered Wnt signaling results in tumorigenesis [10,11]. The members of Wnt family can stabilize β-catenin in an indirect way. β-catenin is an important transcription factor that will transfer into the nucleus to mediate relevant genes influenced by Wnt pathway like c-Myc and Axin2 [10]. C-Myc, as one of the oncogenes, satisfies the demands of rapidly growing cancer cells by up-regulating glycolytic activity [12,13]. Moreover, in addition to lactate dehydrogenase A (LDHA), c-Myc is capable of up-regulating several genes related to glycolysis such as glucose (GLUT1), and hexokinase 2 (HK2). It also significantly promotes the uptake and converse of glucose into lactate, thereby facilitating glycolytic activity [14–16].

TRIM11, an E3 ubiquitin ligase, has been discovered to facilitate various cancers progress [17]. For example, Liu J et al. revealed that TRIM11 accelerated the growth and invasion of hepatoma cell [18]. Yin Y et al. reported that TRIM11 promoted colon cancer progress by enhancing cell viability and inhibiting cell apoptosis [19]. For breast cancer, Wenbo Song et al. uncovered that TRIM11 promoted breast carcinoma cell proliferation via modulating serine/threonine kinase 1/glucose transporter 1 (AKT/GLUT1)-associated glycolysis [20]. Previous studies have reported that Wnt/β-catenin pathway is involved in the development of breast cancer [21,22]. In addition, it is reported that silencing TRIM11 suppressed β-catenin activity through the ubiquitination of Axin1, thereby attenuating lymphoma [23], indicating that TRIM11 possesses regulatory effect on Wnt/β-catenin signaling pathway. Nevertheless, whether TRIM11 regulates Wnt/β-catenin signaling pathway in breast cancer progression remains unclear.

Sex-determining region Y (SRY)-box protein (SOX) family, the testis-determining factor of mammal, consists of approximately 20 SOX proteins, which all have SRY-related high motility group (HMG) domain, the highly conserved DNA-binding sequences in mammals [24]. Recent studies have illustrated that genes in SOX family were tightly correlated with the occurrence and development of diverse cancers, such as hepatocellular carcinoma [25] and squamous-cell carcinoma [26]. SRY-BOX13 (SOX13) as a crucial member of SOX family has been proved to exert essential role in regulating normal and cancer cell properties by mediating Wnt/β-catenin signaling pathway [27]. Previous investigation also found that SOX13 was highly expressed in multiple cancers and contributed to poor prognosis for patients [28,29]. Furthermore, it is reported that SOX13 is able to trigger TRIM11 transcription by binding 18 bp upstream of transcriptional initiation site of TRIM11 promoter and accelerate thyroid cancer [30]. However, the effect of SOX13 on breast carcinogenesis has not been elucidated.

Here, we hypothesized that SOX13 is closely related to the development of breast cancer. Therefore, this work aimed to verify the essential roles of SOX13 in breast cancer progress and to explore the molecular mechanism of SOX13.

## Materials and methods

### Human tissue specimens

Fifty paired breast cancer tissues and their adjacent tissues used in this investigation were collected from Taizhou Municipal Hospital. All patients received mastectomy at this hospital and did not receive chemoradiotherapy or other adjuvant treatment before surgery. All experiments in this study were approved by the Ethics Committee of Taizhou Municipal Hospital (Approval no. 2021LW008) and performed in accordance with the policy of the Declaration of Helsinki. All patients participating in this research have signed informed consent.

## Cell culture

MDA-MB-231, SK-BR-3, ZR-75-30 and BT-474 cells were incubated in Dulbecco’s modified eagle medium (DMEM, L110KJ, Basal Media, China) added with 10% fetal bovine serum at 37°C incubator containing 5% CO_2_. MCF-10A cells were cultured according to a protocol provided in a previous reporter [31].

## Plasmids and RNA inference

To knockdown SOX13, small interfering RNA 5’-GAGAGUAGAUGUCCUAUAA-3’ was purchased from GeneChem Co. Ltd (China). Disordering edition of this sequence was used as control. The above-mentioned sequences were inserted into lentiviral plasmids (pLKO.1), which is operated by GeneChem Co. Ltd. For overexpressing SOX13, full-length sequence of human SOX13 was constructed into pcDNA plasmid.

For transient transfection, plasmids were transfected into cells by using Lipofectamine 2000 (12,566,014, Invitrogen, USA). To establish stable cell lines, the lentiviral plasmid pLKO.1 with shRNA and 2 helper plasmids, psPAX2 and pMD2.G were transfected into HEK293T cells using Lipofectamine 2000. Then, the medium was replaced after 6 h and viral particles in the medium were harvested after 48 h. Next, a 0.45-μm filter was employed to screen the supernatant. Seventy percent confluence MDA-MB-231 cells were infected with the supernatant containing viral particles and 2 μg/ml polybrene (TR-1003-G, Sigma, Germany). Finally, the positive cells were selected by 2 μg/ml puromycin (A1113803, Gibco, USA) [32].

## 3-(4, 5-dimethylthiazol-2-yl)-2, 5-diphenyltetrazolium bromide (MTT) assay

MDA-MB-231 cells were seeded in 96-well plates (5 × 10^3^ cell/mL/well) and were transiently transfected with the indicated vectors for 24 h. Then, 20 μL 5 mg/mL MTT solution (V13154, Invitrogen, USA) was added into each well at 0, 24, 48 and 72 h and incubated with cells for 4 h at 37°C in the dark. Next, MTT solution was replaced with 150 μL dimethyl sulfoxide, and a microplate reader was introduced to record the 590 nm absorbance of cells [32].

## Cell cycle

MDA-MB-231 cells were grown in 6-well plates (5 × 10^5^ cells/well) overnight and were transiently transfected with indicated vectors for 24 h. Then, the cells were harvested in phosphate buffer saline (PBS) solution (B320KJ, Basal Media, China). Seventy percent ethanol was utilized to fix cells in PBS for 2 h on ice, and the cells were washed twice using PBS. Next, the mixture was centrifuged (2000 rpm/min for 5 min), and the supernatant was abandoned. The cells were re-suspended using 500 μl staining buffer (A10798, Invitrogen, USA) and were incubated at 37°C. After 30 min, cells were analyzed with the flow cytometer (CytoFLEX, Beckman Coulter, USA) immediately [33].

## Western blot assay

Total proteins of MDA-MB-231 cells were harvested by using Radioimmunoprecipitation assay buffer (RIPA) lysis buffer (20–188, Millipore, USA), and the concentration was estimated utilizing bicinchoninic acid (BCA) protein assay kit (P0012, Beyotime, China) according to the manufacturer’s protocol. The total proteins were then isolated by 10% sodium dodecyl sulfate polyacrylamide gel electrophoresis (SDS-PAGE) and transferred onto 0.45 μm polyvinylidene fluoride (PVDF) membranes (FFP32, Beyotime, China). Next, the membranes were treated with 3% defatted milk for 40–60 min at room temperature. Subsequently, membranes were incubated with primary antibody SOX13 (18,902-1-AP, Proteintech, USA, 1:1000, Rabbit), lactate dehydrogenase A (LDHA, 3582, Cell Signaling Technology, USA, 1:1000, Rabbit), GLUT1 (ab652, Abcam, UK, 1:1000, Rabbit), hexokinase 2 (HK2) (22,029-1-AP, Proteintech, USA, 1:1000, Rabbit), β-catenin (8480, Cell Signaling Technology, USA, 1:1000, Rabbit), Axin1 (2087, Cell Signaling Technology, USA, 1:1000, Rabbit), CyclinD1 (GB13079, ServiceBio, China, 1:1000, Rabbit), c-Myc (sc-40, Santa Cruz Biotechnology, USA, 1:1000, Mouse), TRIM11 (10,851-1-AP, Proteintech, USA, 1:1000, Rabbit), proliferating cell nuclear antigen (PCNA, sc-56, Santa Cruz Biotechnology, USA, 1:1000, Mouse) and glyceraldehyde-3-phosphate dehydrogenase (GAPDH, 60,004-1-Ig, Proteintech, USA, 1:1000, Mouse) at 4°C for a night. After washing 3 times using Tris Buffered saline Tween (TBST, T9039-10PAK, Sigma, Germany) solution, membranes were incubated with Anti-rabbit immunoglobulin G (IgG), horseradish peroxidase (HRP)-linked Antibody (7074, Cell Signaling Technology, USA, 1:10,000) or Anti-mouse IgG, HRP-linked Antibody (7076, Signaling Technology, USA, 1:10,000) for 1 hour and protein bands were detected with the Hesper chemiluminescence imaging system (GD50401, Monad, China). The levels of proteins were quantified using an imageJ software v 1.8.0 (NIH, USA) [33].

## Immunohistochemistry (IHC)

IHC assay was performed with the guidelines offered in the previous study [34]. Briefly, tissue slices were dewaxed and then were treated with graded ethanol to rehydrate. Next, 10 mM citrate buffer was used to boil the above-mentioned tissue sections for 20 min, and 3% H_2_O_2_ was employed to block the tissues. Subsequently, the tissue slices were incubated with anti-SOX13 (18,902-1-AP, Proteintech, USA, 1:300) antibody, while the control group was treated with immunoglobulin IgG (7074, Cell Signaling Technology, USA, 1:300) overnight at 4°C. Finally, the slices were counterstained with hematoxylin followed by visualizing with 3, 3’-diaminobenzidine [35].

## Glucose consumption, lactate production and adenosine triphosphate (ATP) level detection

In this part, Glucose Assay Kit (F006-1-1, njjcbio), Lactate Assay Kit (MAK064, Sigma, USA) and ATP Assay Kit (A095-1-1, njjcbio, China) were utilized to estimate the glucose consumption, lactate production and ATP level according to the detailed protocol of manufacturer [36].

## Xenograft tumor model

All operations were approved by the Ethics Committee of the Taizhou Municipal Hospital and are in compliance with the policy of the Institutional Animal Care and Use Committee. Briefly, the stably transfected MDA-MB-231 cells were injected into the left flank of nude mice (4–6 weeks, each group had 6 nude mice). The tumor size was recorded at day 7, and it was measured every 7 days for a total of 35 days (The tumorigenesis rate in nude mice was 100%). Finally, the volume of tumor was calculated in accordance with the formula: length × (width^2^/2) [32].

## Statistical analysis

Student’s *t*-test and one-way analysis of variance (ANOVA) were employed to analyze unpaired data and multiple comparisons, respectively, by using GraphPad Prism 6.0. Comparing mean ± deviation (SD) was used to determine statistical significance and P < 0.05 was identified significant (***p < 0.001, ^+++^*p* < 0.001, **p < 0.01, ^++^*p* < 0.01, *p < 0.05, ^+^*p* < 0.05) [35]. All experiments were repeated for at least three times.

## Results

In this study, we assumed that SOX13 exerted crucial roles in breast cancer progression and aimed to verify this hypothesis. In summary, this investigation revealed the increased expression and tumor-stimulative effect of SOX13 on breast cancer progress. In addition, our study expounded that SOX13 was capable of mediating Wnt/β-catenin signaling pathway through TRIM11, which contributed to promote aerobic glycolysis and breast carcinogenesis.

## SOX13 is highly expressed in breast cancer tissues and cells

**Figure 1.**
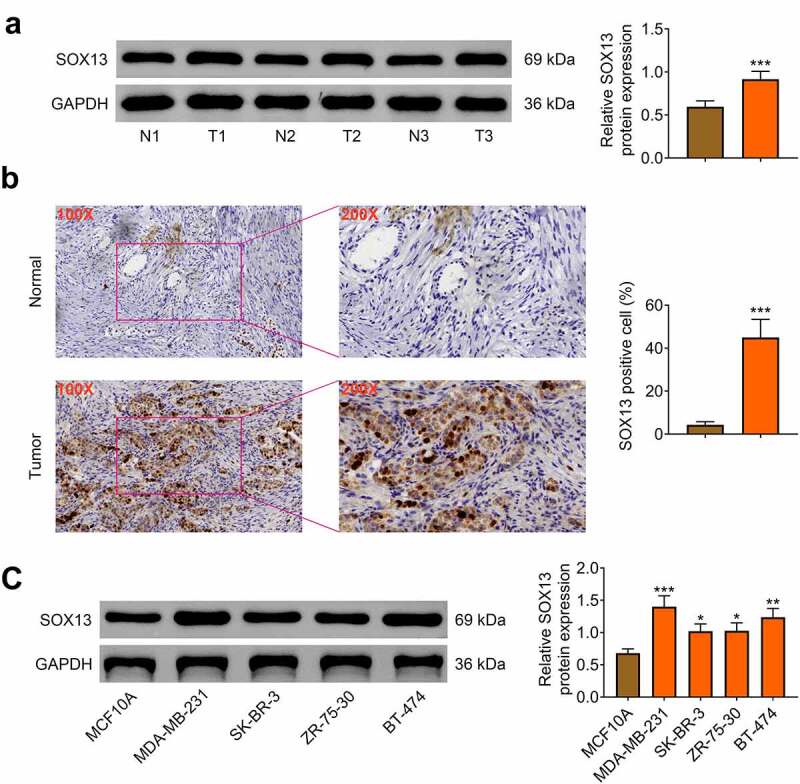
SOX13 is highly expressed in breast cancer tissues and cells. (a) The protein level of SOX13 in breast cancer and normal tissues measured by western blot (***p < 0.001). (b) Immunohistochemical staining of SOX13 detected by IHC (***p < 0.001). (c) The protein level of SOX13 in breast cancer cells and normal cells measured by western blot. Each bar is regarded as the mean ±SD of 3 independent experiments (***p < 0.001, **p < 0.01, *p < 0.05). ***p < 0.001, **p < 0.01, *p < 0.05 versus MCF1A.

First, the protein level of SOX13 in breast cancer and para-carcinoma tissues was measured, and the data illustrated that SOX13 expression was higher in tumor tissues than in para-carcinoma samples (n = 50, ***p < 0.001, Figure 1(a)). Then, immunohistochemical analysis was employed to prove that the protein level of SOX13 was increased in tumor tissues compared with para-carcinoma tissues (***p < 0.001, Figure 1(b)). To explore the role of SOX13 in breast cancer cells, western blot was conducted to estimate the protein level of SOX13 in human epithelial breast cell-line MCF10A and four breast cancer cell lines MDA-MB-231, SK-BR-3, ZR-75-30 and BT-474. As is expected, SOX13 expression at protein level was notably promoted in MDA-MB-231, SK-BR-3, ZR-75-30 and BT-474 cells compared with MCF10A cells (***p < 0.001, **p < 0.01, *p < 0.05, Figure 1(c)). These data showed that SOX13 was obviously up-regulated in breast cancer tissues and cells, implying that SOX13 may be closely associated with the progression of breast cancer.

## SOX13 promotes the proliferation of breast cancer cells

**Figure 2.**
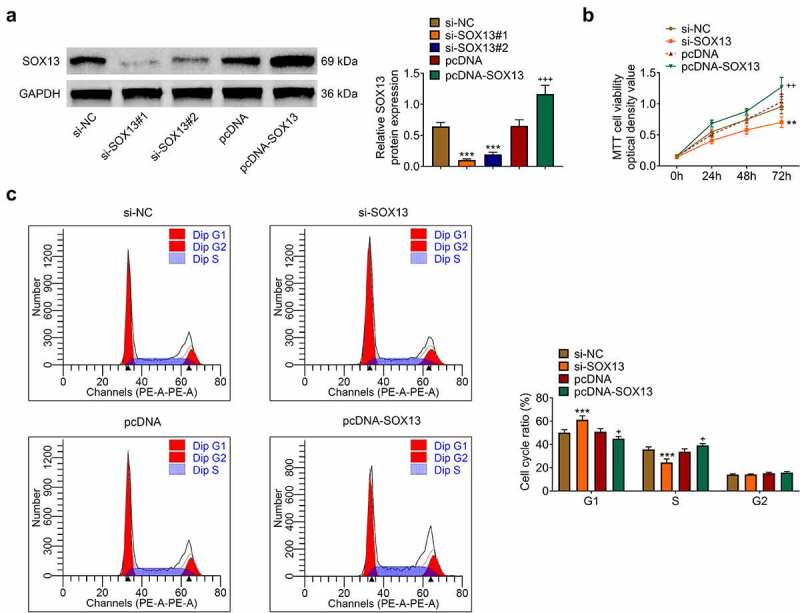
SOX13 promotes the proliferation of breast cancer cells. (a) The protein level of SOX13 measured by western blot (***p < 0.01, ^+++^*p* < 0.001). (b) Cell viability recorded by MTT assay (**p < 0.01, ^++^*p* < 0.01). (C&D) Cell cycle detected by flow cytometry. Each bar is regarded as the mean ±SD of 3 independent experiments (***p < 0.001, ^+^*p* < 0.05). ***p < 0.001, **p < 0.01 versus si-NC group, +++p < 0.001, ^++^*p* < 0.01 versus pcDNA group.

SOX13-targeting siRNA (si-SOX13#1, si-SOX13#2) and corresponding controls (si-NC) were transfected to establish SOX13-knockdown MDA-MB-231 cell lines, while SOX13-expressing plasmids (pcDNA-SOX13) or control plasmids (pcDNA) were introduced to overexpress SOX13. Besides, si-SOX13#1 was chosen for subsequent analyses for its better efficiency (***p < 0.001, ^+++^*p* < 0.001, Figure 2(a)). As shown in 2B, silencing SOX13 dramatically suppressed viability of MDA-MB-231 cells, whereas overexpressing SOX13 accelerated it (**p < 0.01, ^++^*p* < 0.01, Figure 2(b)). The significant effects of SOX13 on MDA-MB-231 cells growth predicted that SOX13 might disturb events related to cell cycle. To verify this hypothesis, we conducted flow cytometry using the above-mentioned cell lines to determine cell cycle progress. The results demonstrated that knockdown of SOX13 notably up-regulated the cell content of G1 stage and down-regulated the cell content of S stage. However, the overexpression of SOX13 decreased the cell percentage of G1 phase and markedly increased the percentage of S phase (***p < 0.001, ^+^*p* < 0.05, Figure 2(c,d)). Thus, SOX13 promoted breast cancer cells proliferation by accelerating G1/S transition of MDA-MB-231.

## SOX13 promotes glycolysis of breast cancer cells

**Figure 3.**
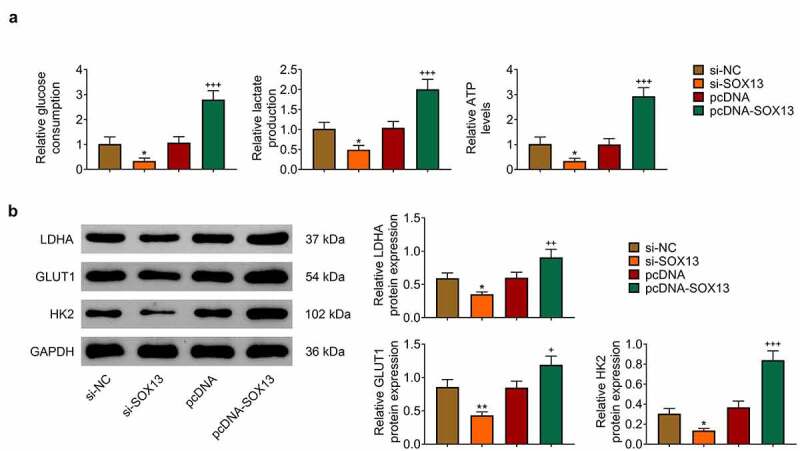
SOX13 promotes glycolysis of breast cancer cells. (a) The glucose consumption, lactate production and ATP level measured by specific assay kit (*p < 0.05, ^+++^*p* < 0.001). (b) The protein level of LDHA, GLUT1 and HK2 detected by western blot. Each bar is regarded as the mean ±SD of 3 independent experiments (**p < 0.01, *p < 0.05, ^+++^*p* < 0.001, ^++^*p* < 0.01, ^+^*p* < 0.05). **p < 0.01, *p < 0.05 versus si-NC group. ^+++^*p* < 0.001, ^++^*p* < 0.01, ^+^*p* < 0.05 versus pcDNA group.

Given that SOX13 promotes glycolysis in multiple myeloma and that glycolysis effectively affects breast cancer growth [37,38], this work investigated whether SOX13 regulates glycolysis in breast cancer cells. Glucose Assay Kit, Lactate Assay Kit and ATP Assay Kit were introduced to reveal that silencing SOX13 dramatically downregulated glucose consumption, lactate production and ATP level, while transfection with pcDNA-SOX13 effectively increased these indexes (*p < 0.05, ^+++^*p* < 0.001, Figure 3(a)). Meanwhile, we analyzed the level of proteins related to glycolysis and discovered that LDHA, GLUT1 and HK2 were reduced by knockdown of SOX13, but dramatically increased by overexpression of SOX13 (**p < 0.01, *p < 0.05, ^+++^*p* < 0.001, ^++^*p* < 0.01, ^+^*p* < 0.05, Figure 3(b)). Taken together, these findings indicated that SOX13 effectively facilitated glycolysis in breast cancer cells.

## SOX13 regulates the Wnt /β-catenin pathway through TRIM11

**Figure 4.**
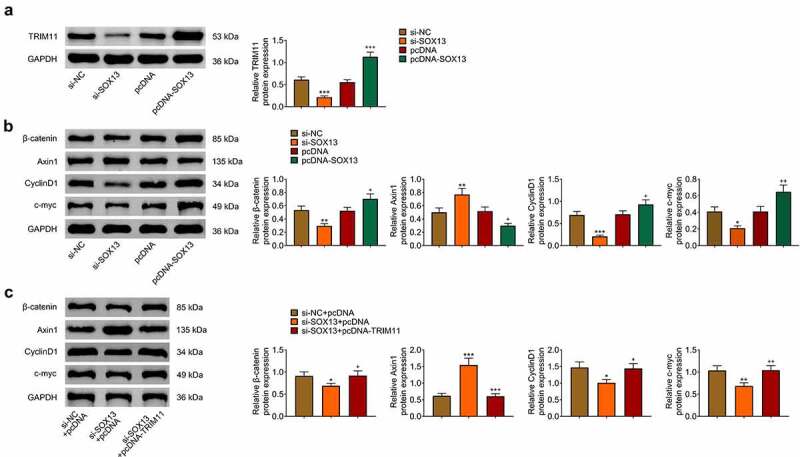
SOX13 regulates the Wnt /β-catenin pathway through TRIM11. (a) The protein level of TRIM11 measure by western blot (***p < 0.001, ^+++^*p* < 0.01). (b) The protein level of β-catenin, Axin1, CyclinD1 and c-myc measured by western blot (***p < 0.001, **p < 0.01, *p < 0.05, ^++^*p* < 0.01, ^+^*p* < 0.05). (c) The protein level of β-catenin, Axin1, CyclinD1 and c-myc treated with TRIM11. Each bar is regarded as the mean ±SD of 3 independent experiments (***p < 0.001, **p < 0.01, *p < 0.05, ^+++^*p* < 0.001, ^++^*p* < 0.01, ^+^*p* < 0.05). ***p < 0.001, **p < 0.01, *p < 0.05 versus si-NC group or si-NC+pcDNA group. ^+++^*p* < 0.001, ^++^*p* < 0.01, ^+^*p* < 0.05 versus pcDNA group or si-SOX13+ pcDNA group.

Previous studies have proved that Wnt/β-catenin signaling pathway exerts vital roles in regulating glycolysis as well as tumorigenesis and TRIM11 mediated the activity of Wnt/β-catenin pathway [10,23,39]. In addition, SOX13 has been found to regulate TRIM11 transcription and affect Wnt signal [27,30]. Therefore, we assumed that SOX13 affected breast cancer and glycolysis by mediating TRIM11/Wnt/β-catenin pathway. As is expected, TRIM11 was decreased by SOX13 knockdown and was obviously increased by SOX13 overexpression (***p < 0.001, ^+++^*p* < 0.001, Figure 4(a)). Additionally, silencing SOX13 significantly reduced the content of β-catenin, CyclinD1 and c-myc, and increased the protein level of Axin1. However, overexpressing SOX13 produced reverse effects (***p < 0.001, **p < 0.01, *p < 0.05, ^++^*p* < 0.01, ^+^*p* < 0.05, Figure 4(b)). Further rescued experiments showed that overexpressing TRIM11 effectively counteracted the effects of SOX13 knockdown on β-catenin, CyclinD1, c-myc and Axin1 (***p < 0.001, **p < 0.01, *p < 0.05, ^+++^*p* < 0.001, ^++^*p* < 0.01, ^+^*p* < 0.05, Figure 4(c)). Collectively, our investigations proved that SOX13 regulated Wnt/β-catenin pathway via TRIM11.

## SOX13 knockdown inhibited tumorigenicity of breast cancer cell *in vivo*

**Figure 5.**
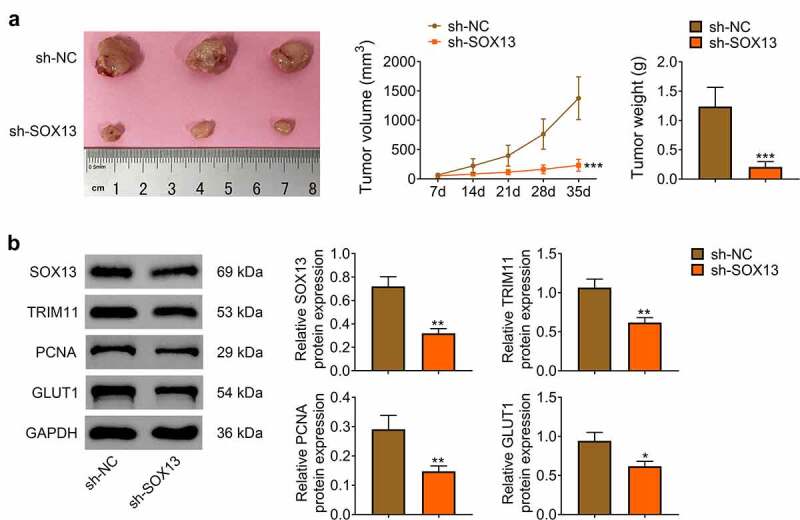
SOX13 knockdown inhibited tumorigenicity of breast cancer cell *in vivo*. (a) The volume and weight of tumor (***p < 0.001). (b) The protein level of SOX13, TRIM11, PCNA and GLUT1 detected by western blot. Each bar is regarded as the mean ±SD of 3 independent experiments (**p < 0.01, *p < 0.05). ***p < 0.001, **p < 0.01, *p < 0.05 versus sh-NC group.

Subsequently, nude mice were employed to determine whether SOX13 affected tumorigenesis *in vivo*. We hypodermically injected MDA-MB-231 cells transfected with sh-SOX13 or sh-NC into nude mice (n = 6 for every group). The tumor formation was recorded at day 7, and it was measured every 7 days for a total of 35 days (The tumorigenesis rate in nude mice was 100%). As presented in Figure 5(a), sh-SOX3 dramatically inhibited tumor growth in vivo with notably decreased volume and weight of tumor (***p < 0.001, Figure 5(a)). Western blot assay illustrated that SOX13 was significantly reduced by sh-SOX13. Meanwhile, silencing SOX13 effectively suppressed the protein level of TRIM11, PCNA and GLUT1, indicating that SOX13 knockdown inhibited growth as well as glycolysis of tumor (**p < 0.01, *p < 0.05, Figure 5(b)). These investigations revealed that silencing SOX13 markedly restrained the formation of tumor by modulating glycolysis.

## Discussion

Breast cancer that exhibits high level of heterogeneity is identified as the most frightening malignancy worldwide [40]. Surgery and chemotherapy as the conventional therapies have not had satisfactory outcomes for patients diagnosed with breast cancer [20]. Hence, further exploration of the complicated molecular network of breast cancer is beneficial to discover accurate prevention and treatment targets for sufferers.

In the present study, we systemically summarized that SOX13 promoted glycolysis and viability of breast cancer cells by activating TRIM11-mediated Wnt/β-catenin signaling pathway. In addition, the results collected from SOX13 knockdown and SOX13 overexpression experiments *in vitro* and *in vivo* were consistent, which confirmed the reliability of our conclusion.

SOX13 is an essential member of SOX family, a collection of transcription factors that play an important role in the progression of cell development. The disorders of SOX genes are verified to be associated with the multiple diseases as both oncogenes and anti-oncogenes [41]. For example, SOX1 was reported to inhibit hepatocellular carcinoma progression by mediating Wnt/β-catenin pathway [42]. On the contrary, SOX10 significantly facilitated and maintained melanoma development [43]. Additionally, SOX13 as a part of SOX family has been found to be involved in diverse cancers, such as colorectal cancer [44], Pancreatic Cancer [45] and gastric carcinoma [28]. Nevertheless, its detailed effects on breast cancer are still unclear. This study discovered that SOX13 was upregulated in breast cancer tissues and cells compared with normal sample. Knockdown of SOX13 dramatically inhibited MDA-MB-231 cells proliferation and arrested cell cycle at G1/S phase, whereas overexpression of SOX13 introduced adverse results. These findings implied that SOX13 might promote breast cancer progression.

Subsequently, we explored the potential mechanisms of SOX13 in breast carcinogenesis, and we found that silencing SOX13 significantly inhibited glycolysis, whereas overexpressing SOX13 accelerated the glycolysis of breast cancer cells. Previous studies have proved that glycolysis is needed for various cancers including breast cancer. It could offer the main energy source under different conditions. Scientists regard this phenomenon as the Warburg Effect, which is characterized by up-regulation of glucose consumption and lactate production [46]. Moreover, the GLUT1, HK2 and LDHA protein levels in cells were increased, indicating the activation of glycolysis [47]. Our investigation revealed that SOX13 inhibition notably decreased the glucose consumption, lactate production and ATP, LDHA, GLUT1 in addition to HK2 level in MDA-MB-231 cells, whereas SOX13 overexpression exhibited reverse effects on these indexes. Additionally, the activation of Wnt/β-catenin has been reported to facilitate the occurrence of glycolysis in cancer cells [48]. TRIM11 was proved to be closely related with breast cancer, and SOX13 was proved to affect various cancers by regulating Wnt/β-catenin pathway [23,49]. Interestingly, our present study confirmed that silencing SOX13 suppressed Wnt/β-catenin pathway activity, while TRIM11 overexpression effectively eliminated these effects, indicating that SOX13 might regulate Wnt/β-catenin pathway via TRIM11. Furthermore, results obtained from xenograft tumor model demonstrated that silencing SOX13 significantly inhibited the tumor growth *in vivo* and reduced TRIM11 and glycolysis-associated proteins expression. These findings suggested that SOX13 exerted vital roles both *in vitro* and *in vivo*. However, how SOX13 mediated TRIM11 and the other downstream genes of SOX13 involved in glycolysis need more investigations in the future.

## Conclusion

In conclusion, this study for the first time revealed that SOX13 promoted breast cancer progression by accelerating TRIM11/Wnt/β-catenin network-mediated glycolysis. These findings strongly suggest that SOX13 as a breast cancer oncogene has the potential to be developed as the biomarker and therapeutic target of breast cancer.

## Supplementary Material

Supplemental Material
