# Supplementary material for: Sex-determining Region Y-box transcription factor 13 promotes breast cancer cell proliferation and glycolysis by activating the tripartite motif containing 11-mediated Wnt/β-catenin signaling pathway
Source: Bioengineered. 2022 May 25;13(5):13033–44. doi: 10.1080/21655979.2022.2073127 (PMC9276007; doi:10.1080/21655979.2022.2073127)

Fig1A---[(1)N1,(2)T1,(3)N2,(4)T2,(5)N3,(6)T3]---SOX13---69 kDa

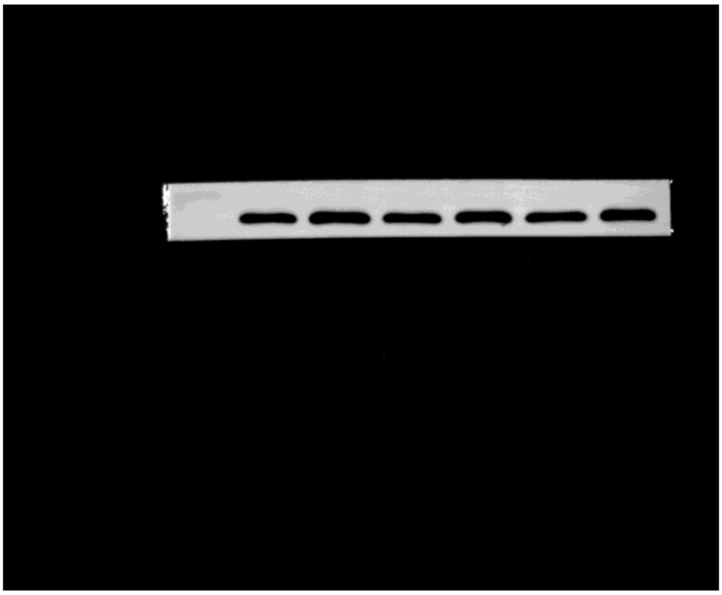

Fig1A---[(1)N1,(2)T1,(3)N2,(4)T2,(5)N3,(6)T3]---GAPDH---36 kDa

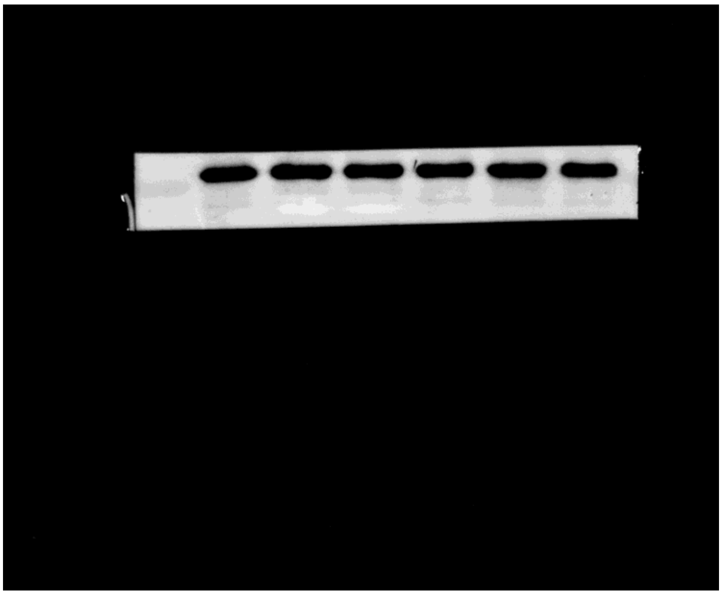

Fig1C---[(1)MCF10A,(2)MDA-MB-231,(3)SK-BR-3,(4)ZR-75-30,(5)BT-474]---
SOX13---69 kDa

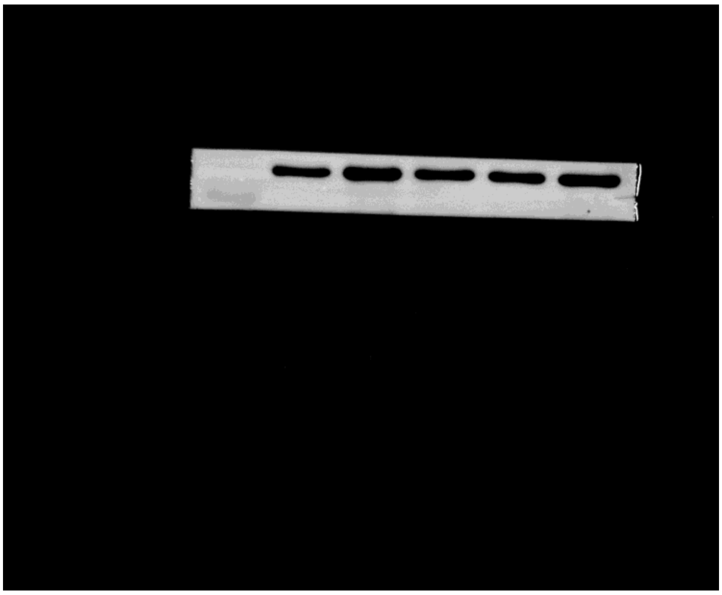

Fig1C---[(1)MCF10A,(2)MDA-MB-231,(3)SK-BR-3,(4)ZR-75-30,(5)BT-474]---
GAPDH---36 kDa

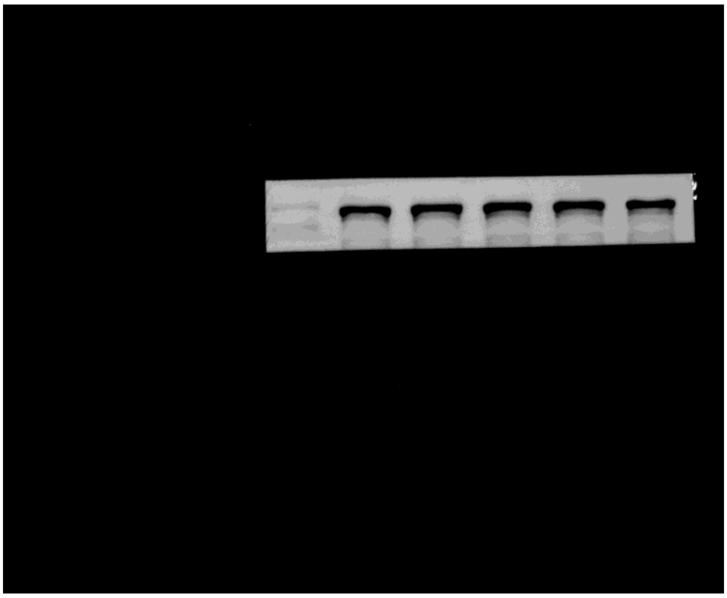

Fig2A---[(1)si-NC,(2)si-SOX13#1,(3)si-SOX13#2,(4)pcDNA,(5)pcDNA-SOX13]---
SOX13---69 kDa

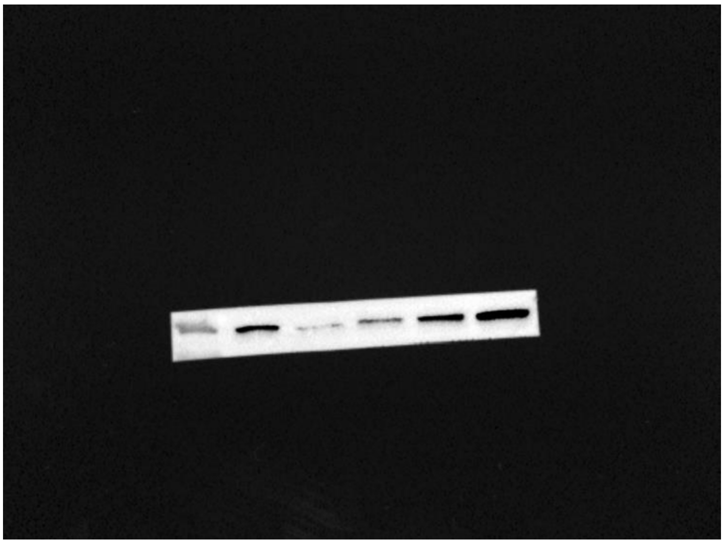

Fig2A---[(1)si-NC,(2)si-SOX13#1,(3)si-SOX13#2,(4)pcDNA,(5)pcDNA-SOX13]---GAPDH---36 kDa

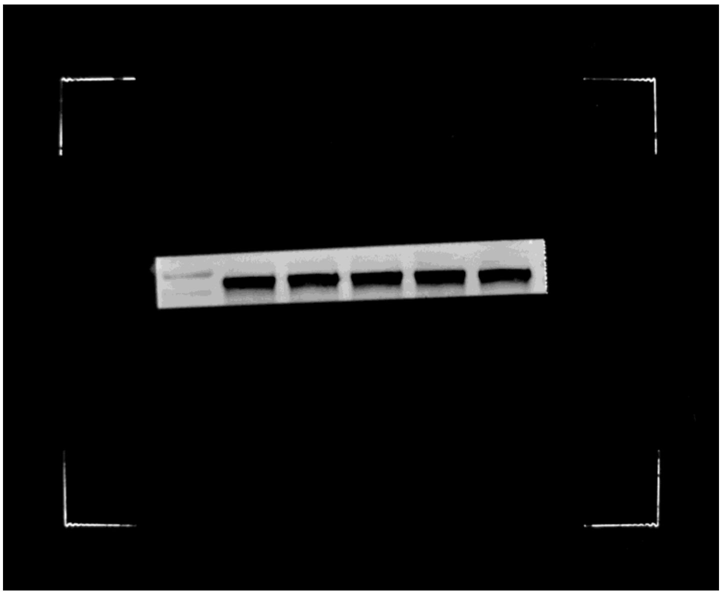

Fig3B---[(1)si-NC,(2)si-SOX13,(3)pcDNA,(4)pcDNA-SOX13]---LDHA---37 kDa

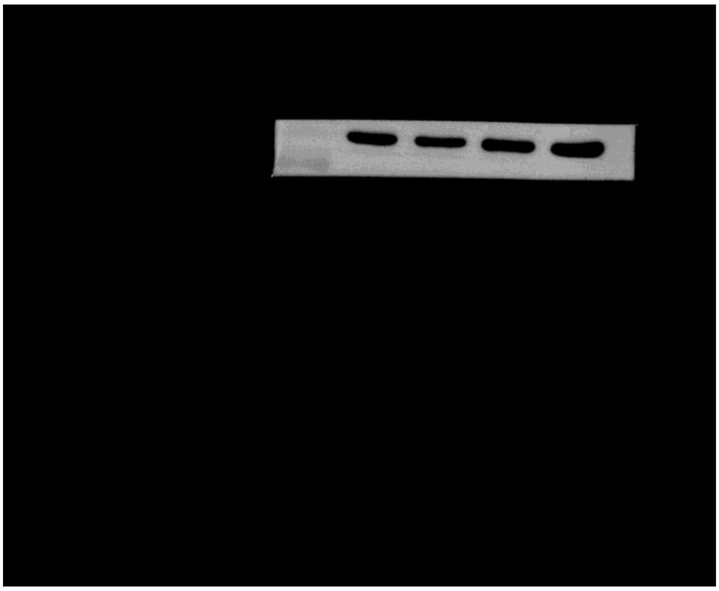

Fig3B---[(1)si-NC,(2)si-SOX13,(3)pcDNA,(4)pcDNA-SOX13]---GLUT1---54 kDa

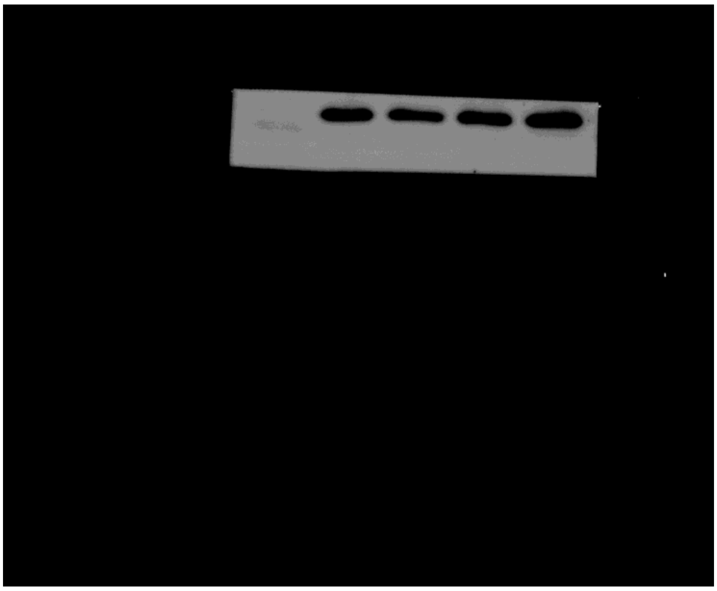

Fig3B---[(1)si-NC,(2)si-SOX13,(3)pcDNA,(4)pcDNA-SOX13]---HK2---102 kDa

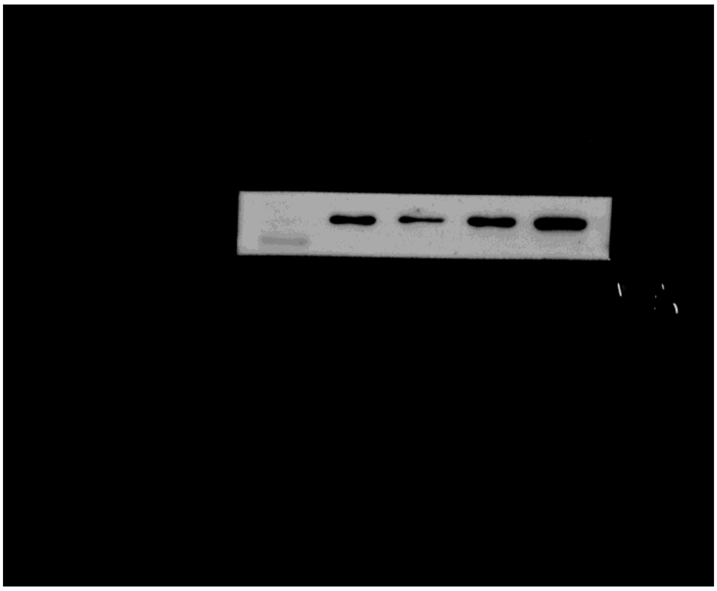

Fig3B---[(1)si-NC,(2)si-SOX13,(3)pcDNA,(4)pcDNA-SOX13]---GAPDH---36 kDa

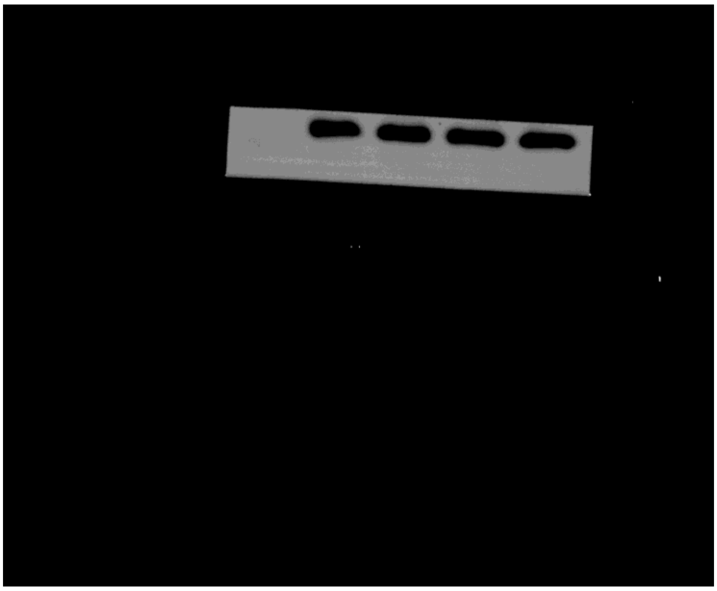

Fig4A---[(1)si-NC,(2)si-SOX13,(3)pcDNA,(4)pcDNA-SOX13]---TRIM11---53 kDa

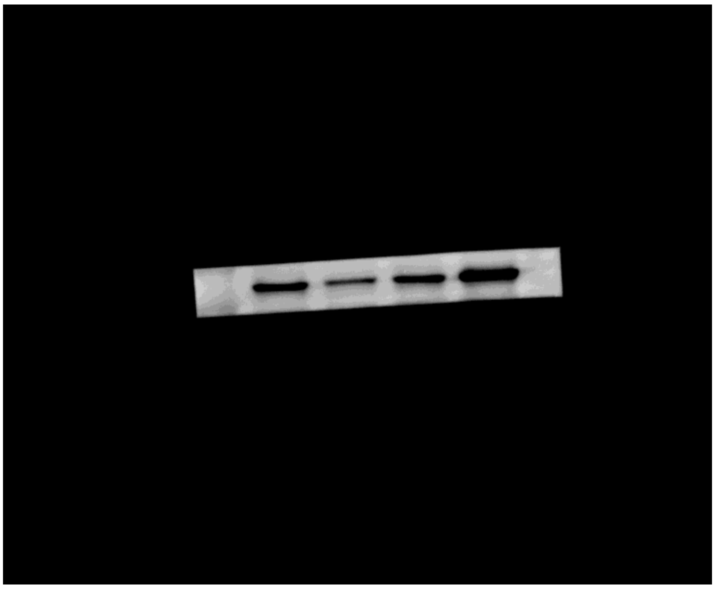

Fig4A---[(1)si-NC,(2)si-SOX13,(3)pcDNA,(4)pcDNA-SOX13]---GAPDH--36 kDa

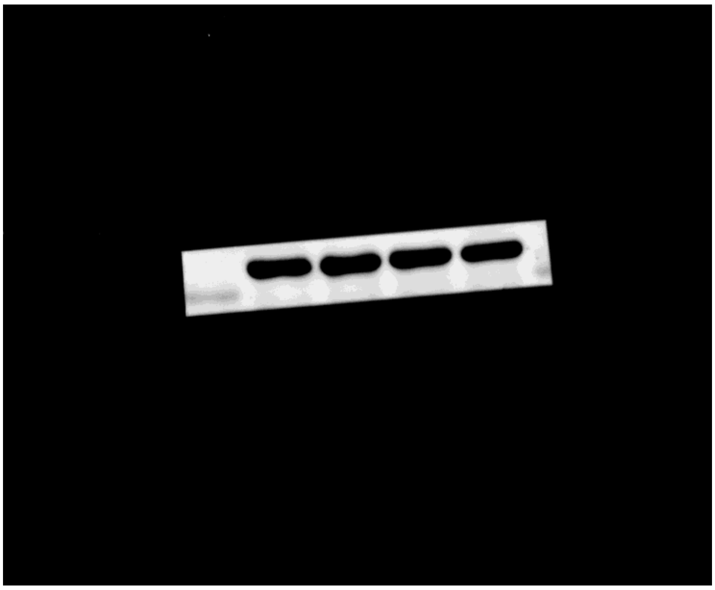

Fig4B---[(1)si-NC,(2)si-SOX13,(3)pcDNA,(4)pcDNA-SOX13]---β-catenin---85 kDa

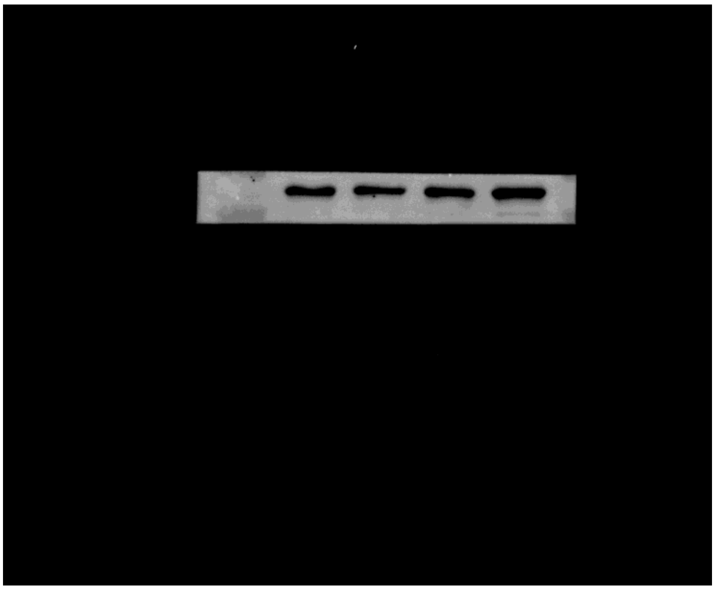

Fig4B---[(1)si-NC,(2)si-SOX13,(3)pcDNA,(4)pcDNA-SOX13]---Axin1---135 kDa

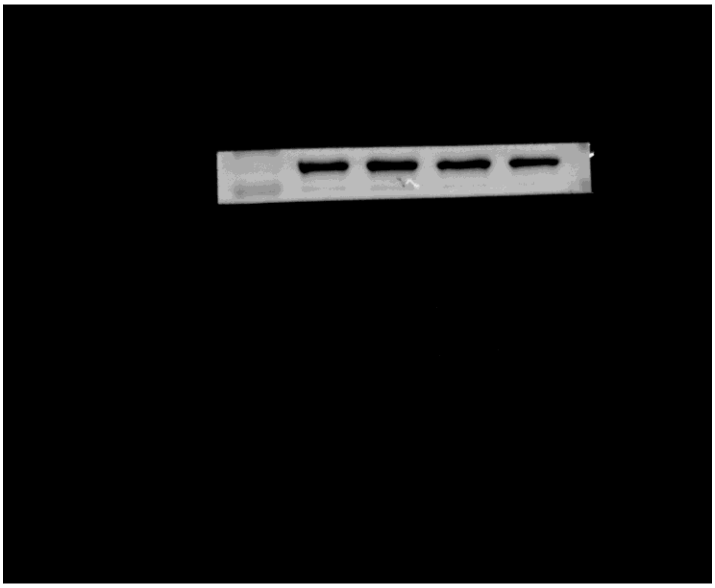

Fig4B---[(1)si-NC,(2)si-SOX13,(3)pcDNA,(4)pcDNA-SOX13]---CyclinD1---34 kDa

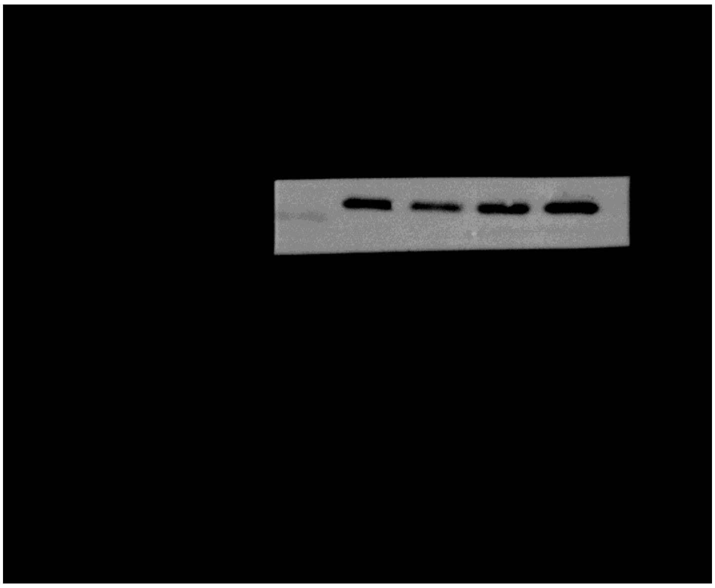

Fig4B---[(1)si-NC,(2)si-SOX13,(3)pcDNA,(4)pcDNA-SOX13]---c-myc---49 kDa

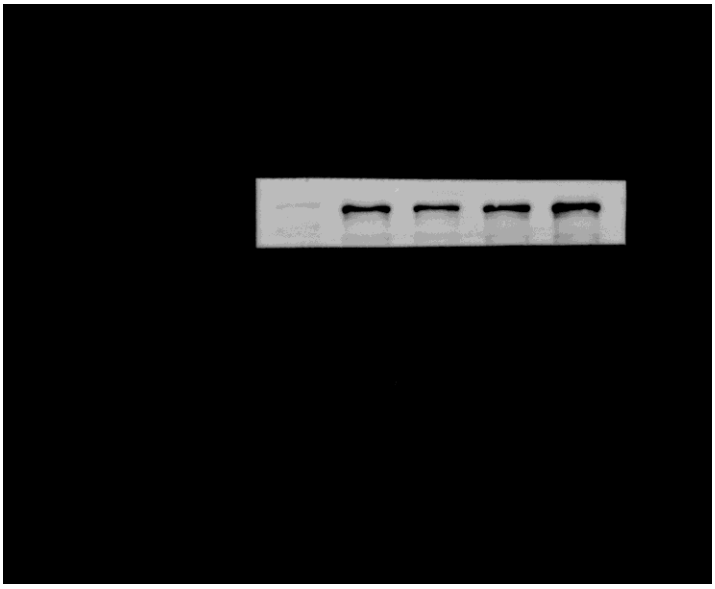

Fig4B---[(1)si-NC,(2)si-SOX13,(3)pcDNA,(4)pcDNA-SOX13]---GAPDH---36 kDa

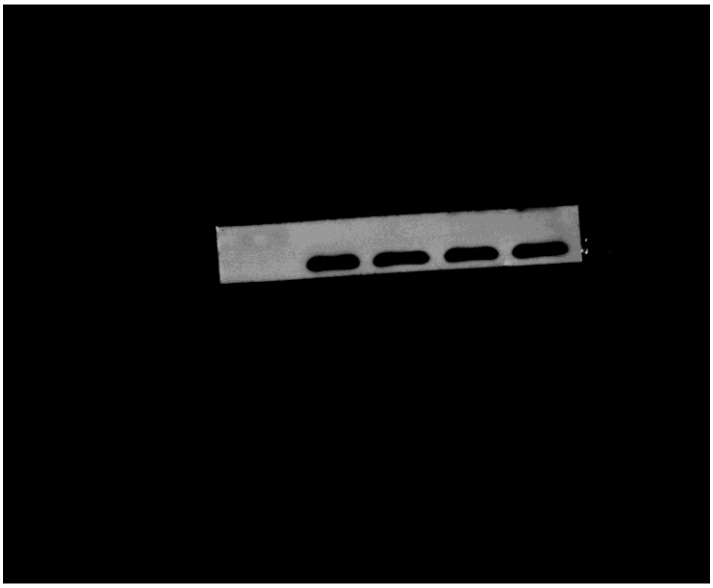

Fig4C---[(1)si-NC,(2)si-SOX13,(3)pcDNA,(4)pcDNA-SOX13]---β-catenin---85 kDa

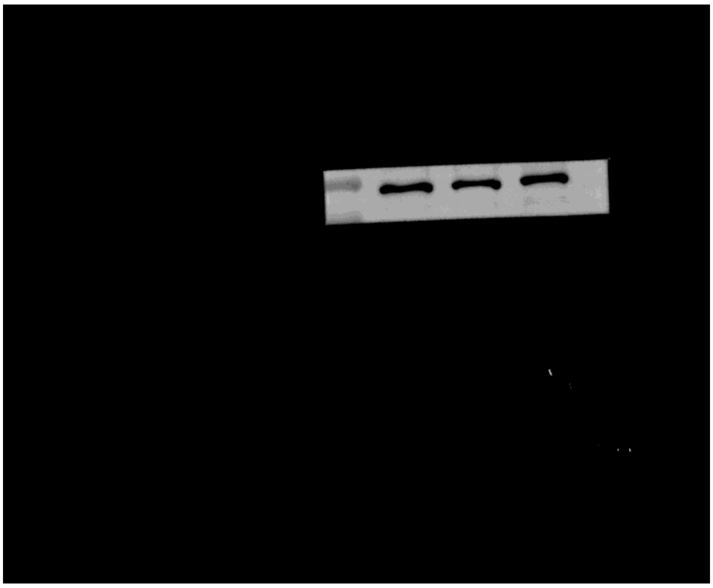

Fig4C---[(1)si-NC,(2)si-SOX13,(3)pcDNA,(4)pcDNA-SOX13]---Axin1---135 kDa

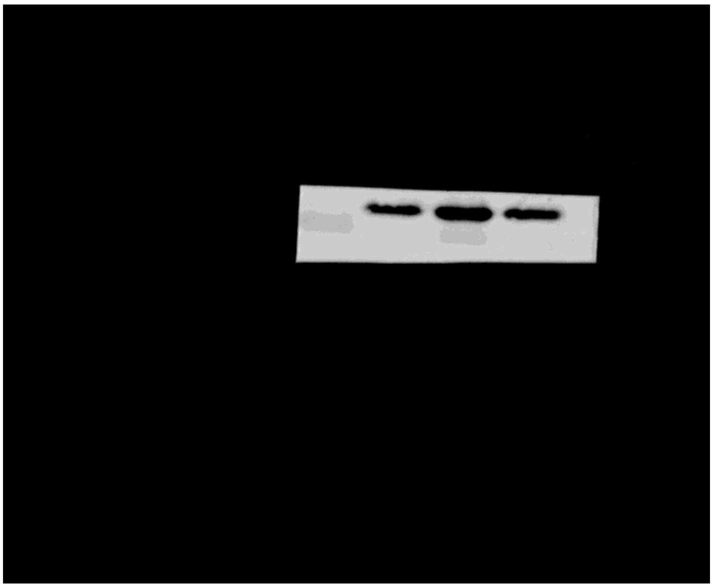

Fig4C---[(1)si-NC,(2)si-SOX13,(3)pcDNA,(4)pcDNA-SOX13]---CyclinD1---34 kDa

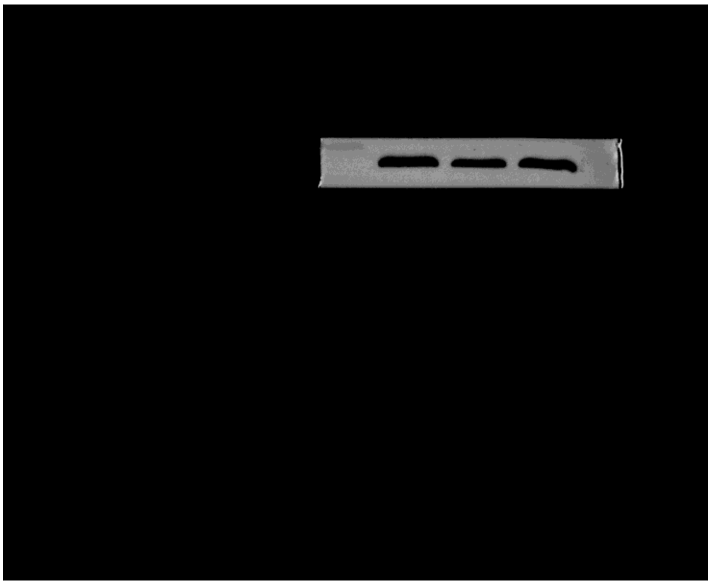

Fig4C---[(1)si-NC,(2)si-SOX13,(3)pcDNA,(4)pcDNA-SOX13]---c-myc---49 kDa

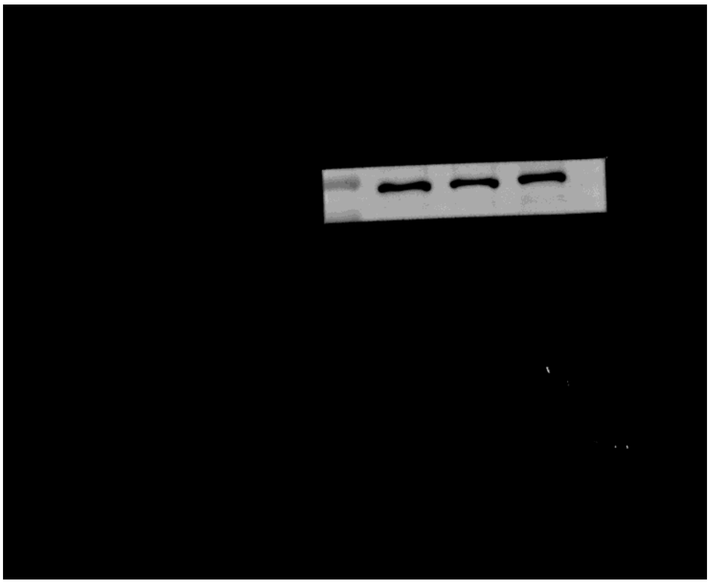

Fig4C---[(1)si-NC,(2)si-SOX13,(3)pcDNA,(4)pcDNA-SOX13]---GAPDH---36 kDa

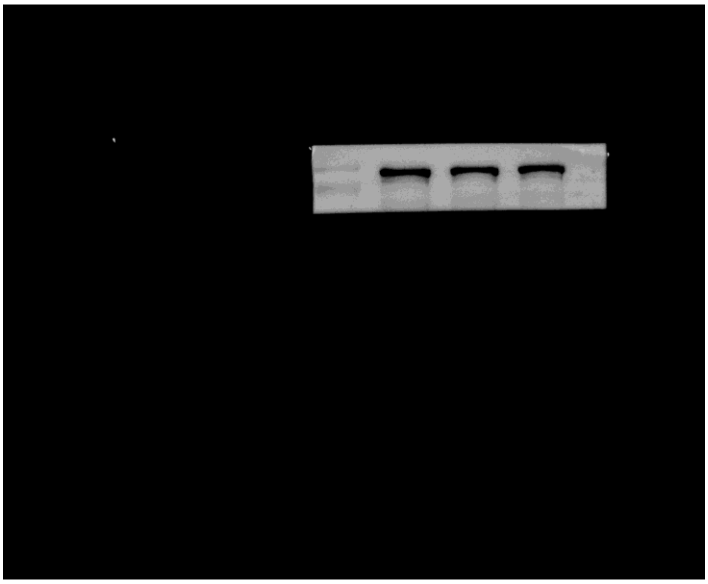

Fig5B---[(1)sh-NC,(2)sh-SOX13]---SOX13---69 kDa

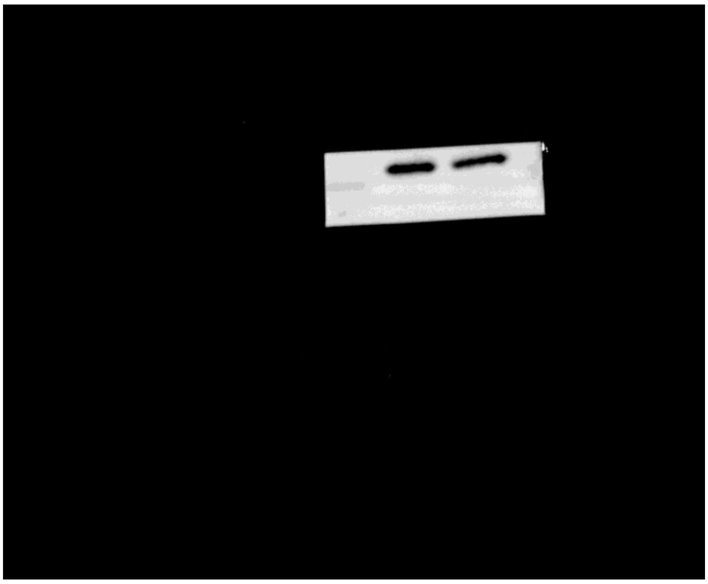

Fig5B---[(1)sh-NC,(2)sh-SOX13]---TRIM11---53 kDa

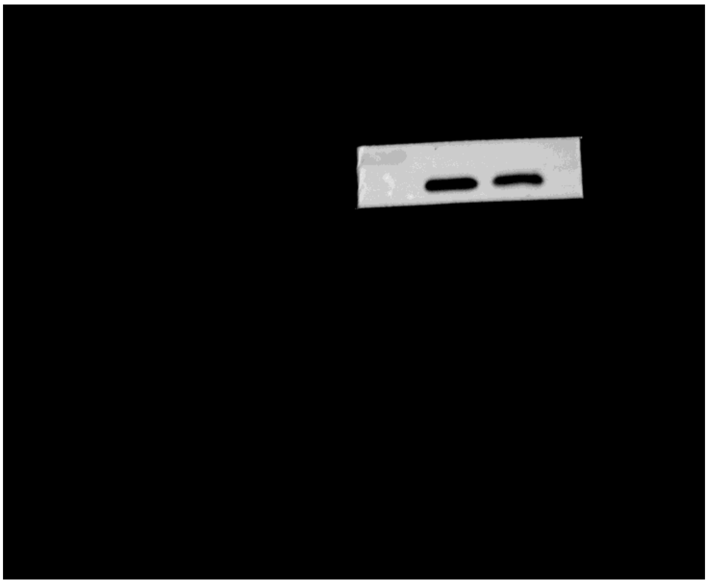

Fig5B---[(1)sh-NC,(2)sh-SOX13]---PCNA---29 kDa

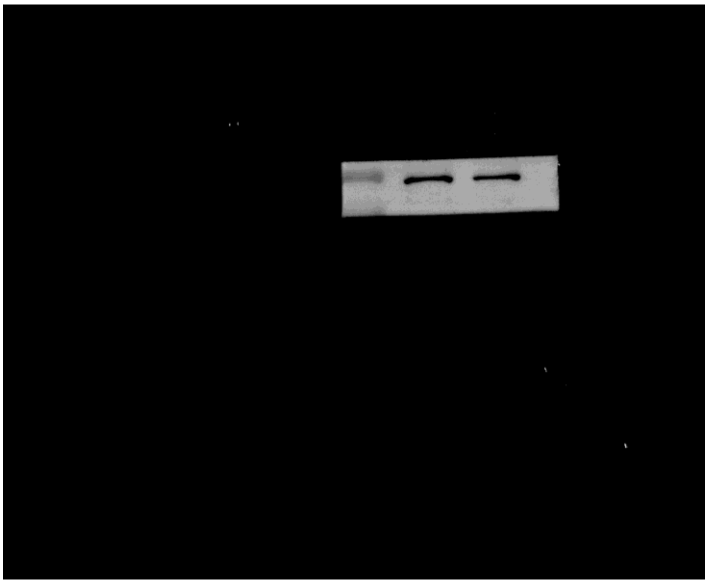

Fig5B---[(1)sh-NC,(2)sh-SOX13]---GLUT1---54 kDa

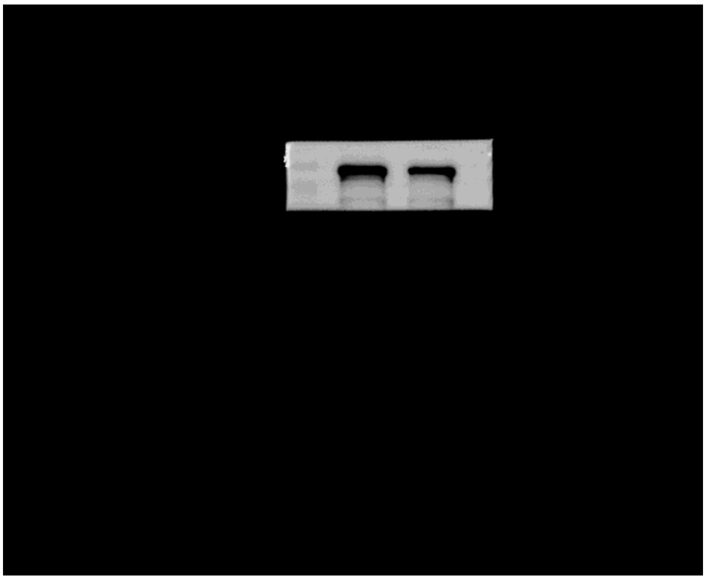

Fig5B---[(1)sh-NC,(2)sh-SOX13]---GAPDH---36 kDa

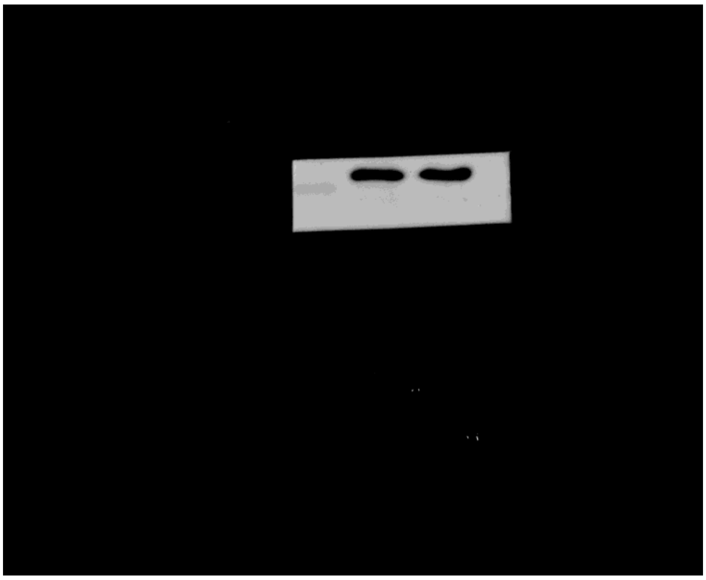

Supplement: Supplemental Material [file KBIE_A_2073127_SM1883.pdf]
